# Wild primate microbiomes prevent weight gain in germ-free mice

**DOI:** 10.1186/s42523-020-00033-9

**Published:** 2020-05-07

**Authors:** Dimitrios N. Sidiropoulos, Gabriel A. Al-Ghalith, Robin R. Shields-Cutler, Tonya L. Ward, Abigail J. Johnson, Pajau Vangay, Dan Knights, Purna C. Kashyap, Yibo Xian, Amanda E. Ramer-Tait, Jonathan B. Clayton

**Affiliations:** 1 Biotechnology Institute, University of Minnesota, 1479 Gortner Avenue, Saint Paul, MN 55108 USA; 2 Johns Hopkins University School of Medicine, Baltimore, MD 21205 USA; 3 Bioinformatics and Computational Biology, University of Minnesota, Minneapolis, MN 55455 USA; 4 Department of Biology, Macalester College, Saint Paul, MN 55105 USA; 5 Primate Microbiome Project, University of Nebraska-Lincoln, Lincoln, NE 68588 USA; 6 Department of Computer Science and Engineering, University of Minnesota, 4-192 Keller Hall, 200 Union St SE, Minneapolis, MN 55455 USA; 7 Enteric Neuroscience Program, Division of Gastroenterology & Hepatology, Departments of Medicine and Physiology & Biomedical Engineering, Mayo Clinic, Rochester, MN 55902 USA; 8 Department of Food Science and Technology, University of Nebraska-Lincoln, Lincoln, NE 68588 USA; 9 Present address: Department of Biology, University of Nebraska at Omaha, Omaha, NE 68182 USA

**Keywords:** Microbiome, Nonhuman primate, Red-shanked douc, Dysbiosis, Westernization, Fecal microbiota transplantation, Obesity, Germ-free mice

## Abstract

**Background:**

The gut microbiome harbors trillions of bacteria that play a major role in dietary nutrient extraction and host metabolism. Metabolic diseases such as obesity and diabetes are associated with shifts in microbiome composition and have been on the rise in Westernized or highly industrialized countries. At the same time, Westernized diets low in dietary fiber have been shown to cause loss of gut microbial diversity. However, the link between microbiome composition, loss of dietary fiber, and obesity has not been well defined.

**Results:**

To study the interactions between gut microbiota, dietary fiber, and weight gain, we transplanted captive and wild douc gut microbiota into germ-free mice and then exposed them to either a high- or low-fiber diet. The group receiving captive douc microbiota gained significantly more weight, regardless of diet, while mice receiving a high-fiber diet and wild douc microbiota remained lean. In the presence of a low-fiber diet, the wild douc microbiota partially prevented weight gain. Using 16S rRNA gene amplicon sequencing we identified key bacterial taxa in each group, specifically a high relative abundance of *Bacteroides* and *Akkermansia* in captive douc FMT mice and a higher relative abundance of *Lactobacillus* and *Clostridium* in the wild douc FMT mice.

**Conclusions:**

In the context of our germ-free mouse experiment, wild douc microbiota could serve as a reservoir for microbes for cross-species transplants. Our results suggest that wild douc microbiota are tailored to diverse fiber diets and can prevent weight gain when exposed to a native diet.

**Graphical abstract:**

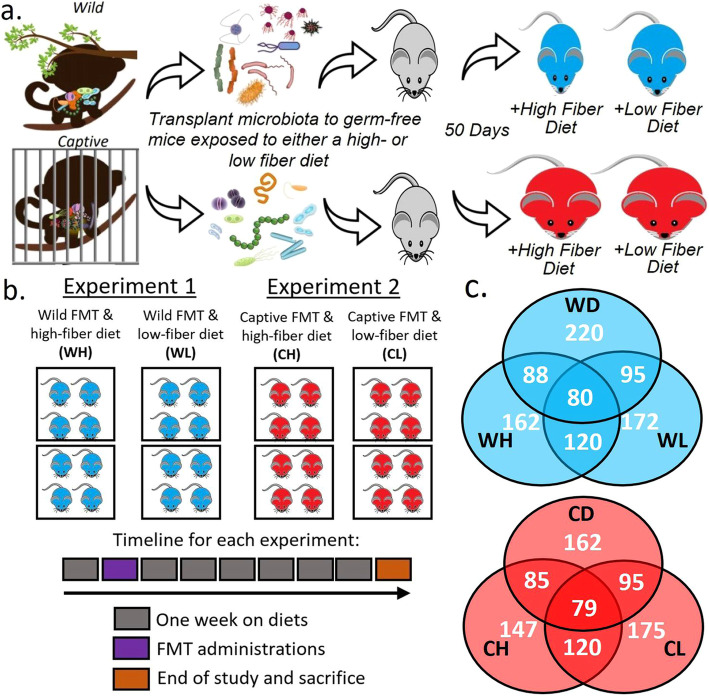

## Background

The human gut microbiome has been linked to numerous diseases, including obesity, diabetes, autoimmune diseases, nonalcoholic fatty liver disease, and colorectal cancer [1, 2]. Gut microbiomes that experience a loss of overall bacterial diversity, increased relative abundance of non-symbiotic bacteria, and loss of beneficial microbes are described as dysbiotic [3]. Due to the integral role the gut microbiome has in the maintenance of health, it is important to identify which bacterial taxa are beneficial, which are contribute to the development of disorders, and how the environment can either protect the microbiome from or drive the microbiome to a dysbiotic state. The contributions of genetics and environmental factors, such as diet or antibiotics, have been studied extensively in humans [4, 5]. The Western lifestyle, for example, tends to include a low-fiber diet and has been linked to obesity, loss of bacterial taxa, and increased relative abundance of phylum Bacteroidetes, versus a high-fiber diet and increased relative abundance of Firmicutes in non-Western parts of the world [6–8].

Wild and captive nonhuman primates (NHPs) provide a unique model to study the relationship between fiber intake and microbiota-associated disorders. Specifically, the red-shanked douc (*Pygathrix nemaeus;* hereafter, douc) are well suited to study these relationships due to their unusually high-fiber diets [9]. Doucs are likely able to metabolize less nutrient-dense diets, largely made up of mature and immature plant parts, due to the cellulolytic microorganisms that colonize the compartments of their GI tract [9, 10]. The microbial populations that inhabit the douc foregut perform digestive processes such as the fermentation of polysaccharides and subsequent production of short-chain fatty acids [9, 11–13]. Captive doucs consume different diets than their wild counterparts [12, 14, 15]. Captive diets lack foods from native, tropical habitats, and instead include nutritional supplements, and commercial primate chow [16]. These captive-primate diets are low-fiber diets, and therefore are not nutritionally comparable to wild diets.

Our previous work showed that NHPs in captivity have gut microbiomes more similar to Western humans than their wild counterparts [17]. In this study, we collected fecal samples from wild and captive doucs and used Fecal Microbiota Transplantation (FMT) to transfer douc gut microbiomes into germ-free mice. We refer to the Wild and Captive douc donor pooled stools used for FMT as Wild Donors (WD) and Captive Donors (CD), respectively. Following transplantation, we exposed the mice to either a high- or low-fiber diet. The study included a total of four experimental groups based on the FMT donor and diet: Captive High (CH), Captive Low (CL), Wild High (WH), and Wild Low (WL). Based on known information about wild and captive douc diet composition, we exposed the microbiomes of WH and CL groups to their native diets (high-fiber and low-fiber, respectively), while challenging the microbiomes of WL and CH groups through exposure to non-native diets (low-fiber and high-fiber, respectively). We hypothesized that the WH microbiome would interact favorably with the high-fiber diet, allowing for the expansion of beneficial microbes, while the CL microbiome would interact unfavorably with the low-fiber diet, resulting in the expansion of microbes associated with a Westernized lifestyle and possibly obesity.

To study the interaction of microbiota and diet, we monitored the health-associated parameters of weight gain and systemic inflammation. For microbial community structure profiling, we performed 16S rRNA gene amplicon sequencing on fecal samples from 32 germ-free mice over a study period of 50 days. The study design allowed us to test for causal relationships between wild or captive microbes and weight gain including possible interactions with diet. Our primary aim was to test whether the gut microbiomes of four wild doucs and two captive doucs shaped by long-term high- and low-fiber diets respectively were either maintained or perturbed when transplanted into germ-free mice exposed to either a high- or low-fiber diet. At the end of the study, we identified key differentially relatively abundant bacterial taxa and physiological responses in these mice resulting from the bacterial colonization post-FMT and diet exposure and observed a strong weight gain phenotype in the mice receiving transplants of captive douc microbiomes.

## Results

**Fig. 1:**
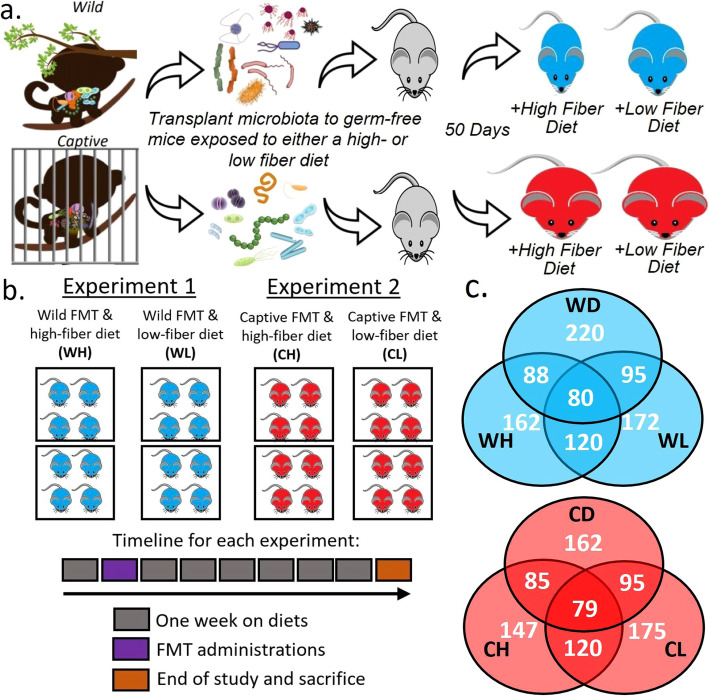
Overview of experimental design (**a**), experimental setup (**b**), and microbiota transfer efficacy (**c**). Germ-free mice were gavaged with either a donor pool FMT from wild (indicated in blue) or captive (indicated in red) doucs and fed a high- or low-fiber diet. Each pair of square boxes indicates a single isolator with four male and four female germ-free mice for a total of four isolators. Reported in (**c**) are total species and overlapping species recovered from fecal samples collected on day 49 prior to sacrifice, as well as from the Wild and Captive FMT donor pools. Abbreviations: Captive High (CH), Captive Low (CL), Wild High (WH), Wild Low (WL), Wild FMT Donor pool (WD), Captive FMT Donor pool (CD).

**Fig. 2:**
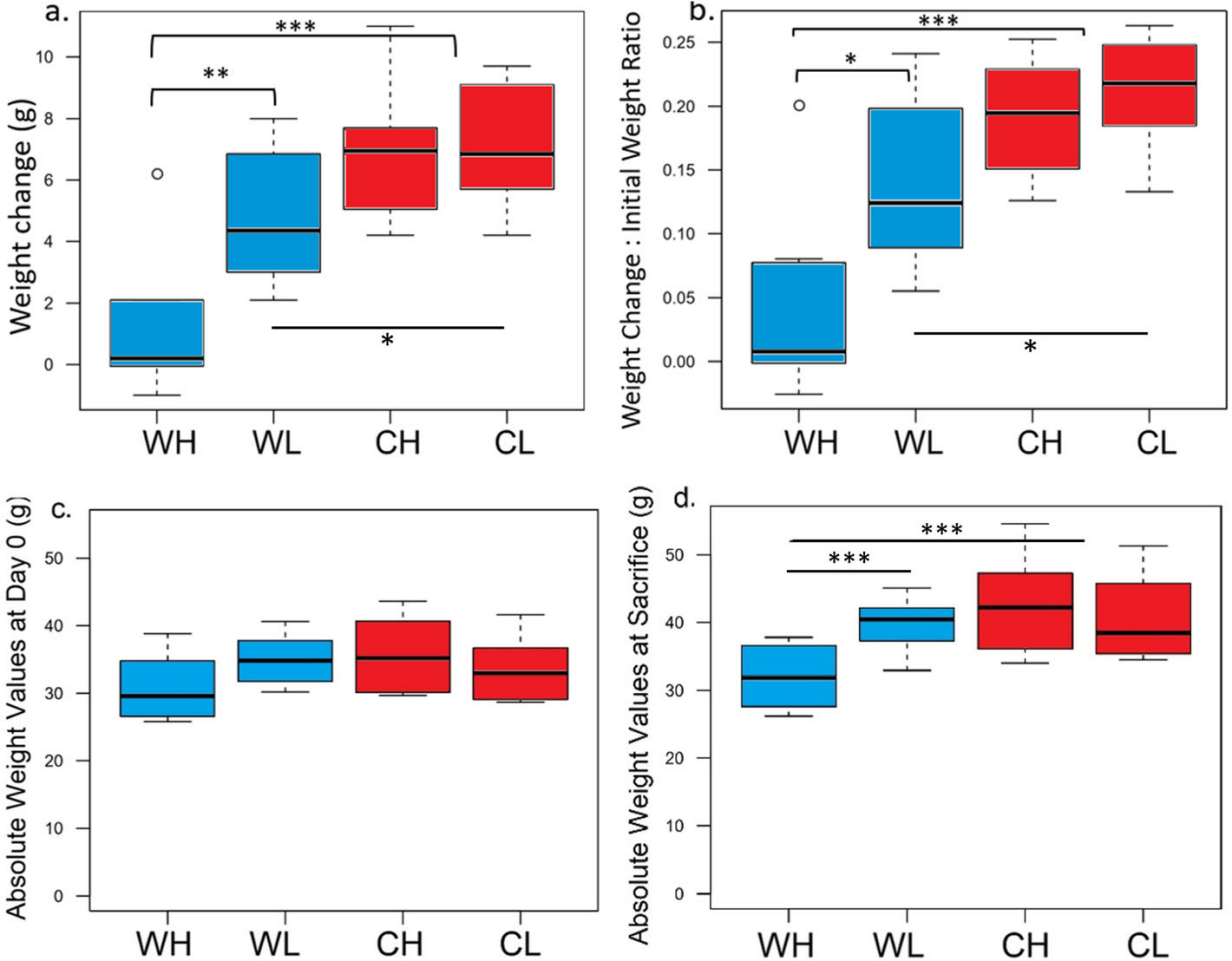
Weight gain in mice from the beginning of the study to sacrifice. Captive donor mice on either diet showed significantly greater weight gain than mice receiving the wild donor microbiota (t-test for group comparisons, two-way ANOVA for diets vs. FMT sources, * p < 0.05; ** p < 0.01; *** p < 0.001).

We were able to transplant a portion of gut microbiota from frozen fecal samples collected from wild and captive NHPs into germ-free mice via a single dosage oral gavage (Fig. 1). We found that mice with wild microbiota exposed to the high-fiber diet did not gain a significant amount of weight by the end of the study compared to their weight before FMT (Fig. 2). Mice with captive microbiota exposed to the low-fiber diet gained the most weight throughout the study. Two-way ANOVA of diets and FMT sources indicated that FMT source is a more significant factor for weight gain using weight change, absolute weight at sacrifice and normalized weight difference as metrics (FMT Source: *p = 1.62e-05, p = 0.00962, p = 1.31e-05* respectively; Diet: *p = 0.0159, p = 0.16173, p = 0.00853* respectively). The Shannon index for alpha diversity showed that evenness increased during the experiment in each group except for Wild High, which maintained its evenness throughout the experiment (Sup. Fig. 1).

**Fig. 3:**
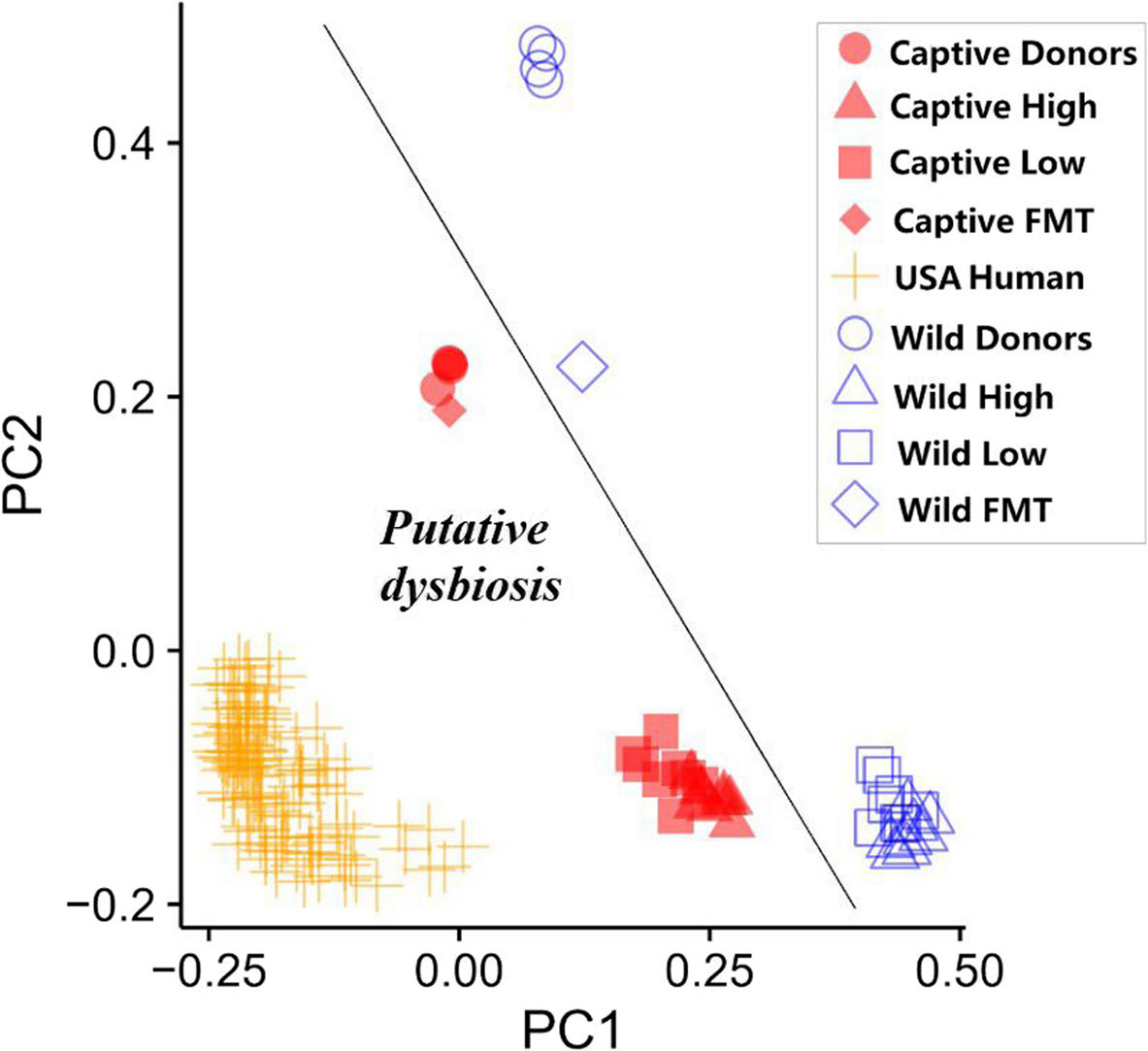
Unweighted UNIFRAC beta diversity per treatment group, individual NHP donors, NHP donor pool used for the FMT, and USA humans. FMT here indicates the pooled fecal samples used to make the single-dose FMT gavage, whereas donors indicate the individual fecal samples used to create the pooled dose. Fecal pellet samples cluster by treatment group. Adonis permutation test showed statistically significant clustering by treatment group (*R*^*2*^ *= 0.24673, p < 0.001)* with the majority of the difference explained by the FMT administered (*R*^*2*^ *= 0.18365*) rather than diet (*R*^*2*^ *= 0.03597*). USA human indicates unweighted UNIFRAC beta diversity of fecal samples from USA individuals from the Global Gut study and fecal samples from this study. The captive groups cluster closer with USA individuals than wild groups (Adonis test *R*^*2*^ *= 0.15861, p < 0.001*). A straight line was added to highlight the difference in clustering between wild and captive samples.

We saw significantly higher alpha diversity with either richness or evenness metrics (*p = 2.48e-14,* ANOVA*, FDR adjusted*) as well as observed OTU counts (*p = 2.69e-08,* ANOVA*, FDR adjusted*) and total bacterial species recovered in mice on a low-fiber diet, groups WL and CL, than those on a high-fiber, groups CH and CL (Sup. Figs. 3 & 4, Sup Table 2). Bray-Curtis non-phylogenetic beta diversity per treatment showed that samples from each treatment group clustered distinctly (Adonis test *R*^*2*^ *= 0.24673, p < 0.001)* with the majority of the difference explained by the microbial community administered (*R*^*2*^ *= 0.18365*) rather than diet (*R*^*2*^ *= 0.03597*). Unweighted UniFrac beta diversity including fecal samples from US individuals in the global gut study by Yatsunenko et al. [8] showed that the captive FMT mice had microbiomes significantly closer to US populations **(**Adonis test *R*^*2*^ *= 0.15861, p < 0.001,* Fig. 3**)**. The Captive Donors clustered with the Captive FMT, however the Wild Donors did not cluster with the Wild FMT. Both FMTs were equally similar to their respective treatment groups, although the treatment groups did not cluster with neither the donors nor the FMTs.

**Fig. 4:**
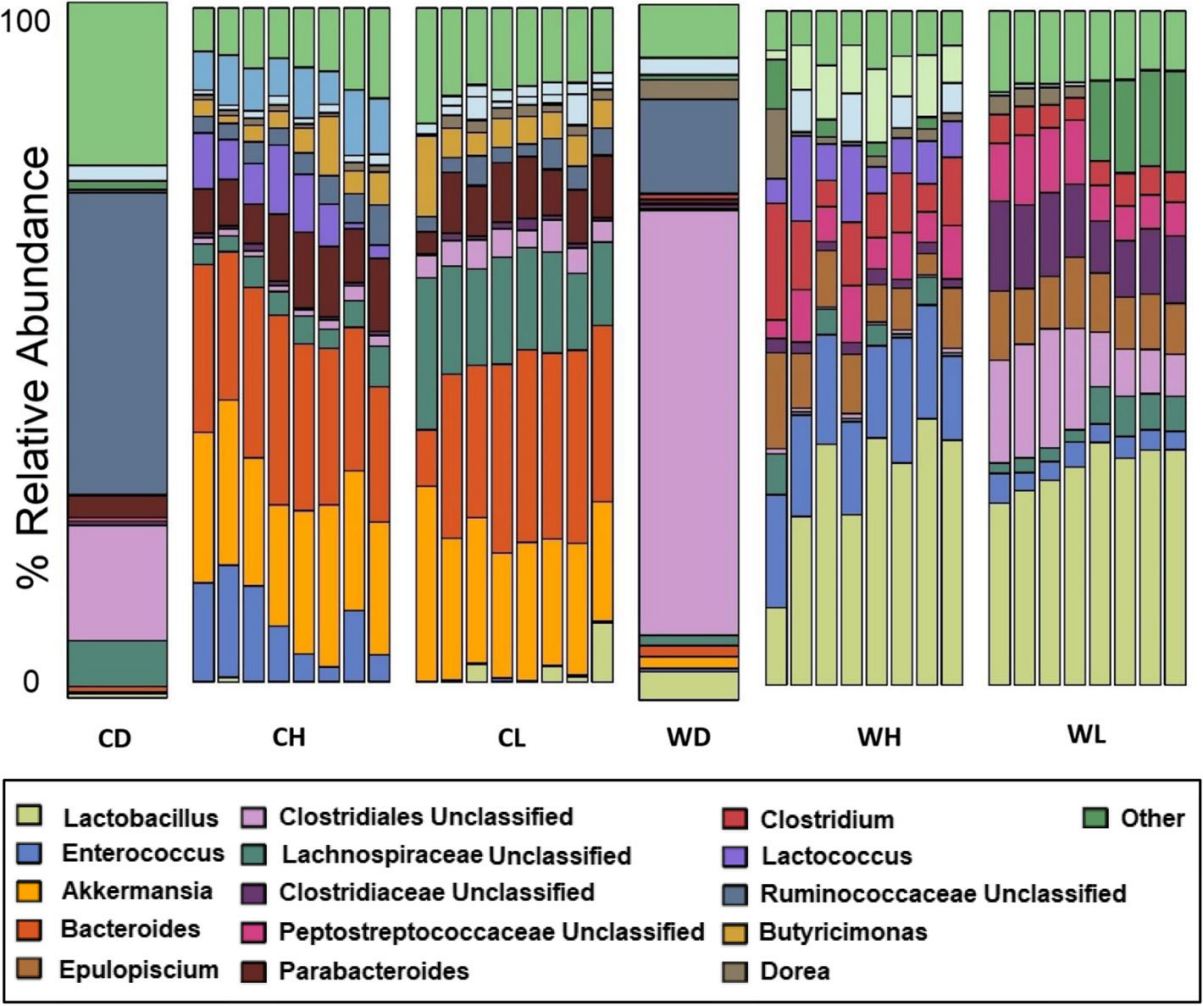
Relative abundance of the top 15 genera in fecal samples immediately before sacrifice. CD and WD bars represent the donor FMTs. All other bars represent 8 mice per group: Captive High (CH), Captive Low (CL), Wild High (WH) and Wild Low (WL). Each color represents a genus in proportion to its relative abundance in each group. We did not find differences between earlier timepoints and the final timepoint with regards to the genera presented here, thus we are showing genera in samples immediately before sacrifice to avoid redundancy.

**Fig. 5:**
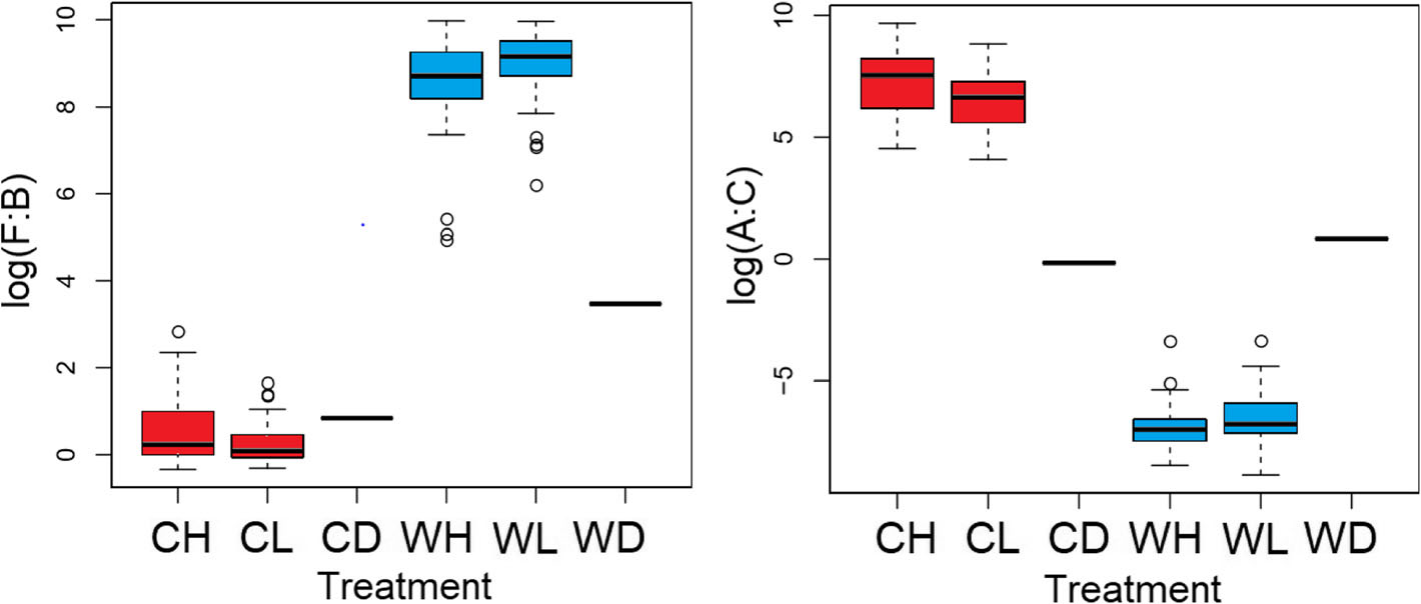
Firmicutes:Bacteroidetes ratio in fecal samples upon sacrifice (left). Wild High and Wild Low groups have a high relative abundance of Firmicutes and no Bacteroidetes. WH and WL thus had a significantly higher F:B ratio than CH and CL (*p < 0.0001,* Wilcox). Akkermansia:Clostridium ratio (right). Captive High and Captive Low groups have a high relative abundance of Akkermansia and little Clostridium. CH and CL groups thus had a significantly higher A:C ratio than WH and WL *(p < 0.0001,* Wilcox*).*

16S rRNA gene amplicon sequencing revealed significant differences in bacterial compositions between the four groups (Fig. 4). Mice receiving the captive donor pool had a higher relative abundance of phylum Bacteroidetes. Mice receiving the wild donor pool had a high relative abundance of phylum Firmicutes yielding a significantly higher Firmicutes:Bacteroidetes ratio than the captive donor pool (*p* < 0.0001, Wilcox, Fig. 5). Analyses of differentiated taxa using a Kruskal-Wallis permutation test with False Discovery Rate (FDR) correction for multiple hypothesis testing revealed distinctly relatively abundant bacterial genera in each group (Table 1). Wild High group had significantly higher *Coprococcus*, *Clostridium*, *SMB53*, *Bacillus and Actinotalea* (Table 2). Wild Low group had significantly higher relative abundance of *Caloramator* and *Paenibacillus*. Captive High group had significantly higher *Akkermansia* and *Turicibacter*. Captive Low had higher *Bacteroides*, *Desulfovibrio* and *Roseburia*. *Enterococcus* and *Lactococcus* were highest in high-fiber diet groups, in both Captive and Wild FMTs (Table 1).

**Table 1:** Differentially relatively abundant genera discovered using Kruskal-Wallis test and False Discovery Rate correction.

Covariate	Genus	Distinctly Abundant	*p*-value
**DIET**	***Enterococcus***	**High-fiber**	*2.57E-13*
	***Lactococcus***	**High-fiber**	*3.17E-19*
**FMT**	***Lactobacillus***	**Wild FMT**	*3.03E-25*
	***Epulopiscium***	**Wild FMT**	*1.13E-25*
	***Clostridium***	**Wild FMT**	*1.05E-25*
	***SMB53***	**Wild FMT**	*3.32E-13*
	***Paenibacillus***	**Wild FMT**	*2.59E-16*
	***Coprococcus***	**Wild FMT**	*5.32E-17*
	***Lysinibacillus***	**Wild FMT**	*1.70E-24*
	***Bacillus***	**Wild FMT**	*1.92E-07*
	***Akkermansia***	**Captive FMT**	*1.05E-25*
	***Bacteroides***	**Captive FMT**	*1.05E-25*
	***Parabacteroides***	**Captive FMT**	*1.05E-25*
	***Christensenella***	**Captive FMT**	*5.98E-27*
	***Roseburia***	**Captive FMT**	*4.20E-16*
	***Butyricimonas***	**Captive FMT**	*1.05E-25*
	***Oscillospira***	**Captive FMT**	*1.36E-25*
**TREATMENT**	***Caloramator***	**Wild Low**	*2.19E-07*
	***Paenibacillus***	**Wild Low**	*1.50E-21*
	***Coprococcus***	**Wild High**	*8.57E-20*
	***Clostridium***	**Wild High**	*4.24E-25*
	***SMB53***	**Wild High**	*3.55E-21*
	***Bacillus***	**Wild High**	*5.24E-18*
	***Bacteroides***	**Captive Low**	*1.51E-24*
	***Desulfovibrio***	**Captive Low**	*1.49E-20*
	***Roseburia***	**Captive Low**	*2.68E-20*
	***Akkermansia***	**Captive High**	*1.24E-24*
	***Turicibacter***	**Captive High**	*2.03E-18*

**Table 2:** Genera distinctly high in the Wild High (lean) group, discovered using two-way ANOVA and False Discovery Rate correction.

Genus	*p* value
***SMB53***	*2.70E-25*
***Bacillus***	*2.11E-21*
***Coprococcus***	*3.60E-08*
***Clostridium***	*1.06E-05*
***Actinotalea***	*2.57E-03*

We compared the taxonomic profiles of fecal samples from each treatment group between 1 week and 6 weeks post-FMT to determine significant differences within each treatment group over time using two-way ANOVA and False Discovery Rate. The WH group had no genera either increasing or decreasing between weeks 1 and 6 of the study post-FMT (summary Table 3). The WL group had an increase of *Coprococcus* (*p = 0.003*) but a decrease in *Enterococcus* (*p = 0.004*), *Epulopiscium* (*p = 0.008*), *Lactococcus* (*p = 0.007*), *Clostridium* (*p = 0.001, FDR adjusted*) and *Paenibacillus* (*p = 0.016*). The CH group had increased *Turicibacter* (*p = 3.48e-07)* but decreased *Coprococcus* (*p = 0.004*). Finally, the CL group had increased *Desulfovibrio* (*p = 0.002*) and decreased *Pseudoramibacter Eubacterium* (*p = 0.0002*). In addition to microbiome analysis, we quantified concentrations of an array of cytokines using blood serum collected at time of sacrifice from all four treatment groups. In a multiplexed quantitative ELISA assay, mice in the WH treatment group had significantly higher levels of circulating cytokines in blood serum compared to the CH treatment group - specifically IL-9, IL-12(P40), IL-13, MCP-1, MIP-1B, MKC and RANTES (Sup. Fig. 4). Mice in CL had significantly higher IL1A levels than WL. Both CH and CL had significantly lower IL-12(P40) levels than WH and WL, respectively. TNF-α in WH was significantly higher than WL and CH but not CL. The cytokines higher in WH comprise a mixture of those positively and negatively associated with inflammation, suggesting that the causal link between captive douc microbiomes and weight gain was not mediated primarily by inflammation.

**Table 3:**
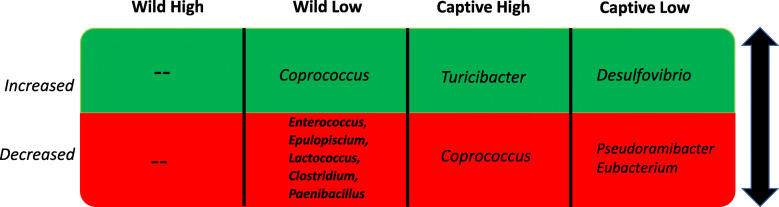
Genera that significantly (*p < 0.05,* ANOVA*, FDR adjusted*) increased (green) or decreased (red) within each treatment group between week 1 and week 6 post FMT. Wild High had no change in bacterial genera.

## Discussion

Our experiment revealed that it is possible to partially transfer microbiota from doucs into germ-free mice using a single dosage of a fecal donor pool derived from frozen fecal samples, and that wild douc microbes partially prevent germ-free mice from gaining weight. In contrast, the captive douc microbiome, which more commonly contains microbes associated with modern humans, caused notable weight gain increase. The gut microbiome of humans living a Westernized lifestyle has been linked to metabolic disorders, including diabetes and obesity. A previous study by Clayton et al. [17] showed that NHPs in captivity have humanized gut microbiomes. Captive doucs lose native microbiota and are colonized by non-native, Western microbiota. Wild doucs are exposed to a diet that contains a diverse fiber content and thus we hypothesized that their microbiota would be challenged by a low-fiber diet. Indeed, the wild microbiota seem to have interacted with the high-fiber diet which resulted in a lean phenotype, whereas an intermediate phenotype resulted when the wild microbiota were exposed to a low-fiber diet. Captive NHPs, as was the case with one of our donors, also tend to be overweight [18, 19]. Our results showed that the weight gain phenotype transferred from captive doucs to mice via the colonization of certain key bacterial taxa in the gut. It is not clear whether the administration of wild microbiota in combination with a high-fiber diet might have also prevented normal weight gain since we were not able to measure weights of mice of identical age exposed to the same diets but not to the FMT. Regardless of exposure to a high or low- fiber diet, mice that received the Captive FMT gained weight, indicating that when weight-influencing bacteria were present in the gut, a high-fiber diet did not have an apparent effect on the phenotype.

A portion of the microbiota in the pooled donor FMTs did not engraft in the treatment groups (Supp. Table 1). This happened possibly because donor samples were frozen, and some bacteria could not survive passage through the gastrointestinal tract, or they were not able to colonize due to key wild host and environmental factors that were absent in this study. It is interesting to note that fewer bacterial species were engrafted from the Wild FMT (WD), than the Captive FMT (CD) (see microbiota transfer efficacy in Supp. Table 2). In addition, when looking at the beta-diversity clustering of donors with FMTs, it is apparent that the Wild Donors did not cluster with the Wild FMT, instead the Wild FMT lies in between the Wild Donors and Wild treatment groups. Captive Donors, on the other hand did cluster with the Captive FMT (Fig. 5). It is likely that some of the microbiota in the Wild Donors are more sensitive and less robust for transplantation or were weakened after the process of freeze-thawing and FMT gavage preparation.

A number of studies have shown a link between increased alpha diversity when consuming a high-fiber diet [20]. In this study, we found that alpha diversity, total bacterial species discovered and observed OTUs were higher in mice exposed to the low-fiber diet, and it was equivalent between FMT groups, which does not correspond to previous findings with human microbiota [21]. This might be partly because the low-fiber diet has a simpler formula composed mostly of sucrose, cornstarch, cellulose, casein and corn-oil. It is possible that this formula of more universal nutritional sources in the context of our experiment and tested microbiota could sustain a greater spectrum of douc microbiota. We also acknowledge the limitations of de novo and open reference methods for picking OTUs. While our goal was to capture the full diversity of the native communities, it is possible that despite filtering for spurious hits these methods may result in inflated observed OTU counts.

An increasing Shannon index for alpha diversity over time indicates that evenness increased during the study for the non-lean groups WL, CH, and CL. It was also found that in these groups there were key bacterial genera that either significantly increased or decreased during the course of the study. The WH group, however, had a steady evenness throughout the experiment, as well as no significant changes in key bacterial genera, suggesting that a more stable microbiome, in addition to presence of key diverse fiber-interacting microbes, contributed to a lean phenotype. Particularly interesting was the decrease of Clostridium in WL, which was significantly higher in WH than any other group (*p = 1.06E-05)*, indicating there may be some unknown beneficial Clostridium strains that colonize and interact favorably with a high-fiber diet but decrease over time when exposed to a low-fiber diet.

Beta diversity analysis showed that samples from each group clustered together indicating that there are bacterial taxa that drive distinctions between treatment groups. Kruskal-Wallis permutation test revealed certain genera that were higher in each group (Table 1). Specifically, we found a higher relative abundance of *Bacteroides* in Captive FMT, which is one of the most common genera in the Western microbiome, and no traces of *Bacteroides* in Wild FMT. The Wild FMT groups primarily carried Firmicutes, suggesting that increased relative abundance of Firmicutes genera with the absence of *Bacteroides* might have contributed to a healthier state. Captive FMT also had a high relative abundance of *Akkermansia muciniphila* (~ 20%), a mucin-degrading bacterium capable of subsisting on the intestinal mucus layer. A previous study used *Akkermansia muciniphila* as treatment to reverse high-fat diet-induced weight gain and metabolic disorders [22]. In their study, a high relative abundance of *Akkermansia muciniphila* inversely correlated with body weight in humans and mice, which does not correspond with our findings, suggesting that the effects of this strain might be more nuanced when combined with NHP microbiota.

In our study, weight gain was primarily influenced by the FMT and not the diet as indicated by both Bray-Curtis non-phylogenetic beta diversity and two-way ANOVA on weight data. Interestingly, both wild and captive FMT donor pools had a similar relative abundance of *Akkermansia muciniphila*. However, it only colonized in Captive FMT mice and became extinct in the Wild FMT mice. Previous work has shown that *Akkermansia muciniphila* relative abundance is inversely correlated with *Clostridium difficile* relative abundance [23]. Interestingly, the *Akkermansia*:*Clostridium* ratio was significantly higher in Captive FMT treatment groups, and low in Wild FMT treatment groups (*p < 0.001* Wilcox, Fig. 5). However, an equal intermediate ratio was observed in both Donor FMTs. This might suggest the reason why *Akkermansia* did not colonize in Wild FMT mice is partly due to the presence of *Clostridium*, which was in significantly lower abundance in the Captive FMT groups (Kruskal Wallis *p = 1.05E-25* FDR adjusted). Further exploration is required to thoroughly understand this relationship and the extent of its impact on host health.

## Conclusions

Our study demonstrates that captive douc microbiomes cause weight gain under high-fiber and low-fiber diets in germ-free mice. Wild douc microbiomes moderated this weight gain phenotype on a low-fiber diet and caused no weight gain when combined with a high-fiber diet. This suggests that bacteria such as *Coprococcu*s, *SMB53* and *Bacillus*, *Actinotalea,* and *Clostridium* that were significantly more relatively abundant in the Wild-High group might have an effect in preventing weight gain in combination with a high-fiber diet. These results also show that the captive douc microbiome, a more similar microbiome to the modern human gut microbiome, can cause weight gain regardless of dietary fiber content, while the wild douc microbiome can partially prevent mice from gaining weight when exposed to a low-fiber diet. These findings confirm that microbiome transplant alone is sufficient to prevent or cause weight gain, and further that there is a microbiome-dependent effect on the weight fluctuating nature of certain diets, supporting a clinical role for manipulation of the microbiota.

## Methods

### Study subjects and sample material

Fecal samples from wild NHPs (doucs) inhabiting Son Tra Nature Reserve, Da Nang, Vietnam and captive NHPs (doucs) housed at the Philadelphia Zoo were used as the starting material for FMT. The fecal samples of these donors were frozen upon collection, and ultimately sequenced and characterized [17]. We thus had a microbiome profile of the donors prior to initiating the study. The study was conducted at the germ-free mouse facility of the Mayo Clinic in Rochester, MN. Thirty-two germ-free 6-weeks old Swiss Webster mice were exposed to either a high-fiber or low-fiber diet (Fig. 1). Feed was provided ad libitum; mice were checked daily and feed added to feeders as needed. A total of 4 isolators were used, each isolator had 2 cages of mice divided by gender, one with 4 females and the other with 4 males. The study was conducted in two separate experiments with 16 mice in each one. The first experiment used 2 isolators with 4 male and 4 female mice in each isolator all gavaged with the wild douc stool samples. Similarly, the second experiment also used 2 isolators with 4 male, 4 female mice in each isolator all gavaged with the captive douc stool samples. In each experiment, one isolator was fed the high fiber diet and the other one was fed the low fiber diet. Mice were weighed at the start of the study and immediately preceding sacrifice with CO_2_ asphyxiation. After a week of acclimation, mice received the donor microbiota via gavage of fecal slurry. Fecal samples were collected weekly after FMT. At day 50 on the experimental diets, mice were sacrificed. Blood, and stool samples were collected. All samples were placed in RNA*later* solution and frozen immediately at − 80 °C. Blood samples were immediately centrifuged to separate serum at 15000 rpm for 10 min, and then frozen at − 80 °C. Frozen serum samples were analyzed for cytokines and chemokines using the Milliplex Mouse Cytokine/Chemokine Magnetic Bead Premixed 25 Plex Kit (Millipore Sigma Catalog # MCYTOMAG-70 K-PMX).

### Colonization of germ-free mice with NHP microbiota

Fecal samples from wild and captive doucs were used to create 2 master donor pools for the transplantation experiments. We decided to pool donor samples in order to capture and test in vivo the maximum number of microbiota possible against the two diets. For the captive donor pool, 4 samples from 2 captive doucs, 1 male and 1 female, from 2 different timepoints were used for a total of 4 donor stool samples. The female donor was 23.3% more overweight than an average female douc [24, 25]. The male donor had a normal average weight at the time of collection. For the wild donor pool, samples from 4 wild doucs, 2 males and 2 females, were used for a total of 4 donor stool samples. For each donor pool, 2.5 mL aliquots from each stool sample were homogenized in a sterile 10 mL pre-reduced 1x PBS solution in the anaerobic chamber. Germ free Swiss Webster mice maintained in flexible film gnotobiotic isolators in the Mayo Clinic Germ Free Mouse facility were used in the study. All mouse experiments were approved by Mayo Clinic IACUC. Each mouse received a one-time 200 μl of FMT gavage that is 50% PBS and 50% stool sample. This yielded an effective dose of 100 μl of douc stool per mouse. A 500 μl aliquot of each donor pool was saved for 16S rRNA gene amplicon sequencing to get a bacterial profile of the combined donors.

### Diets

We based our diet selection on a previous microbiota-accessible carbohydrate diet study by Sonnenburg et al. [26]. We used Pico-Vac 5061 (LabDiet, St. Louis, MO) for our high-fiber diet due to lower carbohydrate levels and more diverse fiber content. We used Teklad Diet TD.86489 (Envigo, Somerset, NJ) as our low-fiber diet (see Supp. Figs. 5 & 6 for diet composition details of low-fiber and high-fiber diets, respectively). Both diets were ordered in irradiated isolator compatible vacuum sealed packaging to eliminate the introduction of bacterial contaminants in the gnotobiotic isolators. The 5061 diet is considered a “high-fiber” diet because it has a diverse fiber content of 4.7% crude fiber (up to 6%) which includes 16.4% neutral detergent fiber (cellulose, hemi-cellulose and lignin) and 6% acid detergent fiber (cellulose and lignin). The TD.86489 diet is considered a “low-fiber” diet because its single source of fiber is cellulose (5%) and its main source of carbohydrates (61.7%) is sucrose (53%) and cornstarch (47%). Percentages indicate ratios by weight of feed. The low-fiber diet’s energy value is 3.7 kcal/g, whereas for the high-fiber diet it is 4.07 kcal/g.

### Bacterial 16S rRNA gene PCR amplification and next-generation sequencing

Samples were lysed using the PowerMag Microbiome Lysis Solution and DNA was extracted using a PowerMag Microbiome RNA/DNA Isolation Kit (QIAGEN Catalog #27500–4-EP) from fecal, cecum, and jejunum samples. The bacterial 16S rRNA gene was amplified using Dual-Index Microbiome Amplification at the University of Minnesota Genomics Center (UMGC) [27]. The protocol uses 515F and 806R primers, which flank the V4 hypervariable region of the 16S rRNA gene (515F: TCGTCGGCAGCGTCAGATGTGTATAAGAGACAGGTGCCAGCMG CCGCGGTAA; 806R: GTCTCGTGGGCTCGGAGATGTGTATAAGAGACAGGGACTACHVGGGTWTCTAAT). First reaction involved 25 cycles using the Meta_V4_515F/Meta_V4_806R primer pair. After the first round of amplification, PCR 1 products are diluted 1:100 and 5 ul of 1:100 PCR 1 is used in the second PCR reaction which uses different combinations of forward and reverse indexing primers for a total of 10 cycles. Amplicons were sequenced using Illumina MiSeq paired end sequencing at UMGC. Pooled size-selected sample was denatured with NaOH, diluted to 8 pM in Illumina’s HT1 buffer, spiked with 20% PhiX, and heat denatured at 96C for 2 min immediately prior to loading. A MiSeq 600 cycle v3 kit was used to sequence the sample. Nextera adapter sequences were used for post-run trimming (Read 1: CTGTCTCTTATACACATCTCCGAGCCCACGA GACNNNNNNNNATCTCGTATGCCGTCTTCTGCTTG, Read 2: CTGTCTCTTATACACATCTGA CGCTGCCGACGANNNNNNNNGTGTAGATCTCGGTGGTCGCCGTATCAT). Sequences were cleaned and converted from FASTQ to FASTA format using SHI7 v0.92 with parameters *--allow_outies F - t 8 -s T -filter_q 34 -trim_q 32* [28].

### Data analysis

We used QIIME Open Reference Operational Taxonomic Unit (OTU) Picking to pick OTUs and calculate alpha and beta diversity metrics using the SHI7 quality-controlled sequence reads [28]. Singleton OTUs were excluded during open reference picking in QIIME using *pick_open_reference_otus.py* and default parameter *--min_otu_size 2* which drops OTUs with less than 2 counts. QIIME version 1.9.1 was used, all samples were rarefied at 14,000 sequences, and Pynast alignment failures were omitted from all subsequent analyses [29]. NINJA-OPS 1.5.1 [30] closed reference OTU picking with Greengenes 97% identity was used for comparison of this study’s taxonomic profiles to the global gut study [8]. Observed OTUs refer to the OTU counts calculated using tables that were not collapsed by taxonomy. For all taxonomic and differential analyses, taxa tables were collapsed to genus or species levels and rare taxa with less than 20 counts were dropped except for when reporting total recovered species.

### Statistics

Kruskal-Wallis permutation and two-way ANOVA tests with False Discovery Rate *p*-value correction revealed genera that were higher in each FMT, diet, and treatment group. Two-way ANOVA was used for weight data differences by FMT and diet, and t-test for pair-wise group weight comparisons. Adonis permutation test was used to determine significant clustering of samples using beta diversity. Linear regression and a groupwise average of the test statistic of the spearman correlation were used to determine shifts in Shannon Index alpha diversity in each treatment group over time. All statistics, meta-analyses and plotting were conducted using R statistical package (version 3.4.0).

## Supplementary information

**Additional file 1:Figure S1.** Shannon index for alpha diversity (evenness) 1 week, 3 weeks and 6 weeks after FMT. Shannon index significantly increased in the captive high, captive low, and wild low groups but remained stable in wild high, the leanest group. Linear regression and a groupwise average of the test statistic of the Spearman correlation were used to determine shifts within treatment groups.

**Additional file 2: Figure S2.** Shannon index for alpha diversity (evenness) in each treatment group. High-fiber groups had significantly lower Shannon index than low-fiber groups (*p = 2.48e-14,* ANOVA*, FDR adjusted*), but differences between wild and captive FMTs were not statistically significant.

**Additional file 3: Figure S3.** Observed OTUs in each treatment group. High-fiber groups had significantly lower observed OTUs than low-fiber groups (*p = 2.69e-08,* ANOVA*, FDR adjusted*), but differences between wild and captive FMTs were not statistically significant.

**Additional file 4: Figure S4.** Detectable circulating blood cytokine and chemokine levels after sacrifice, from top left to bottom right: G-CSF, IL-1A, IL12(P40), IL-12(P70), IL-1B, IL-9, IL-10, IL-13, IL-15, RANTES, MCP-1, IP-10, MKC, MIP-2, TNF-A, MIP-1A, MIP-1B. All concentrations calculated in pg/mL Bars in green are showing WH, in light green WL, in blue CH and in purple CL. Concentrations of GM-CSF, IFN-G, IL-2, IL-4, IL-5, IL-6, IL-7 and IL-17 in most samples were lower than detectable level. Two-tailed t-test used (ns: no significant; * p < 0.05; ** p < 0.01; *** p < 0.001).

**Additional file 5:Figure S5.** Low-fiber diet composition by manufacturer.

**Additional file 6:Figure S6.** High-fiber diet composition by manufacturer.

**Additional file 7:Table S1.** Bacterial taxa in donor FMTs that are missing in their respective treatment groups.

**Additional file 8:Table S2.** Microbiota transfer efficacy as indicated by the total bacterial species & mean observed OTUs identified in fecal samples collected on day 49 and compared to donor FMT.

## Data Availability

All nonhuman primate fecal sample sequencing data are deposited at the European Bioinformatics Institute under the project number *PRJEB11414*. All mouse fecal sample sequencing data are deposited at the European Bioinformatics Institute under the project number *PRJEB32832*. Mouse fecal samples extracted DNA and RNA are available at the UMGC under project name *Knights_Project_034.*

## Abbreviations

NHP: Nonhuman primate
FMT: Fecal Microbiota Transplantation
CH: Captive High
CL: Captive Low
WH: Wild High
WL: Wild Low
WD: Wild Donors FMT
CD: Captive Donors FMT
OTU: Operational Taxonomic Unit
FDR: False Discovery Rate
GF: Germ-free
PMP: Primate Microbiome Project
UMGC: University of Minnesota Genomics Center
