# Supplementary figures and images for: Wild primate microbiomes prevent weight gain in germ-free mice
Source: Anim Microbiome. 2020 May 7;2:16. doi: 10.1186/s42523-020-00033-9 (PMC7807445; doi:10.1186/s42523-020-00033-9)

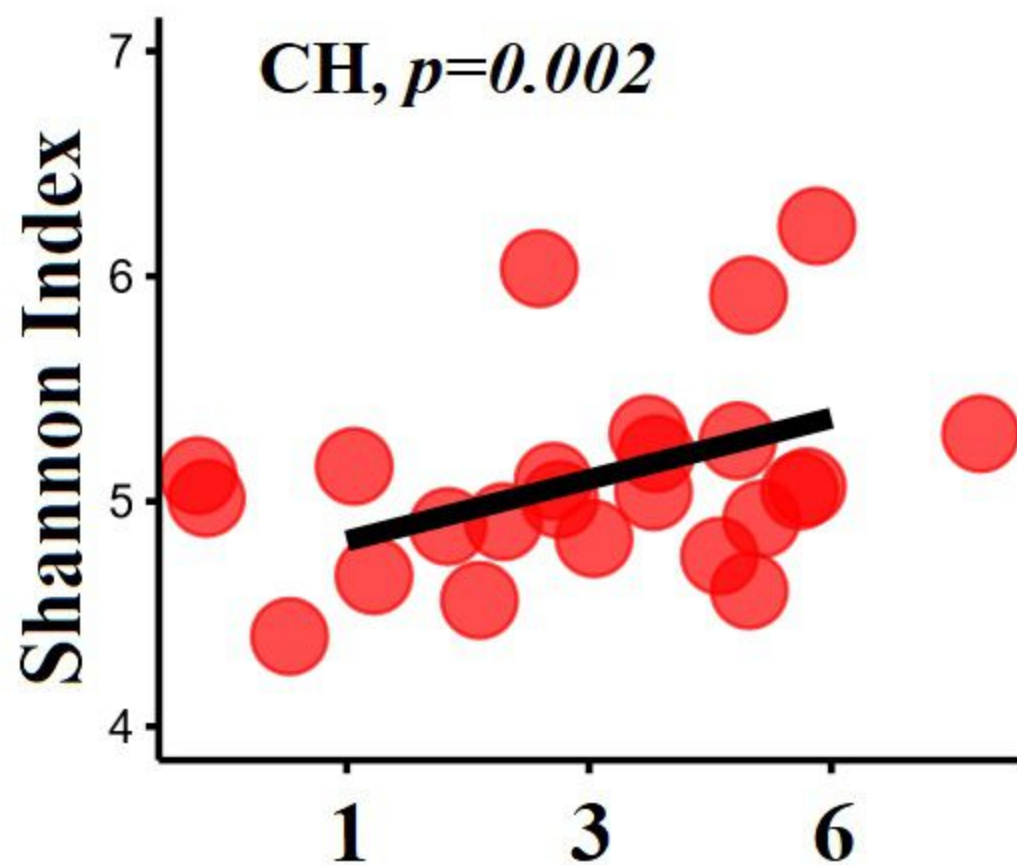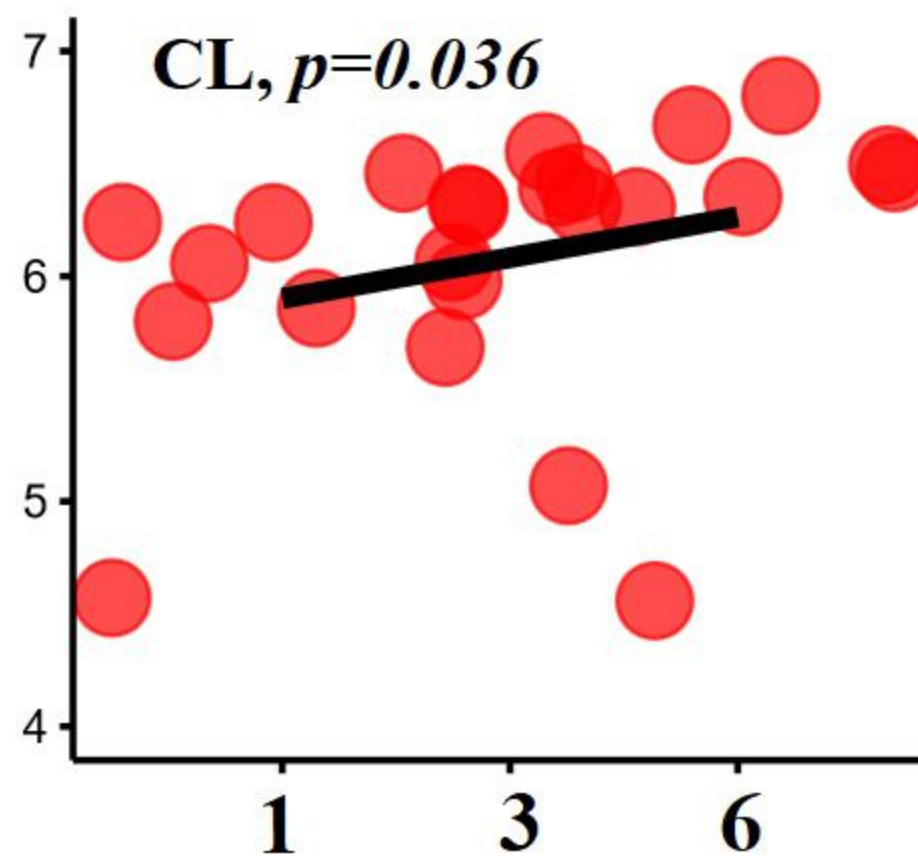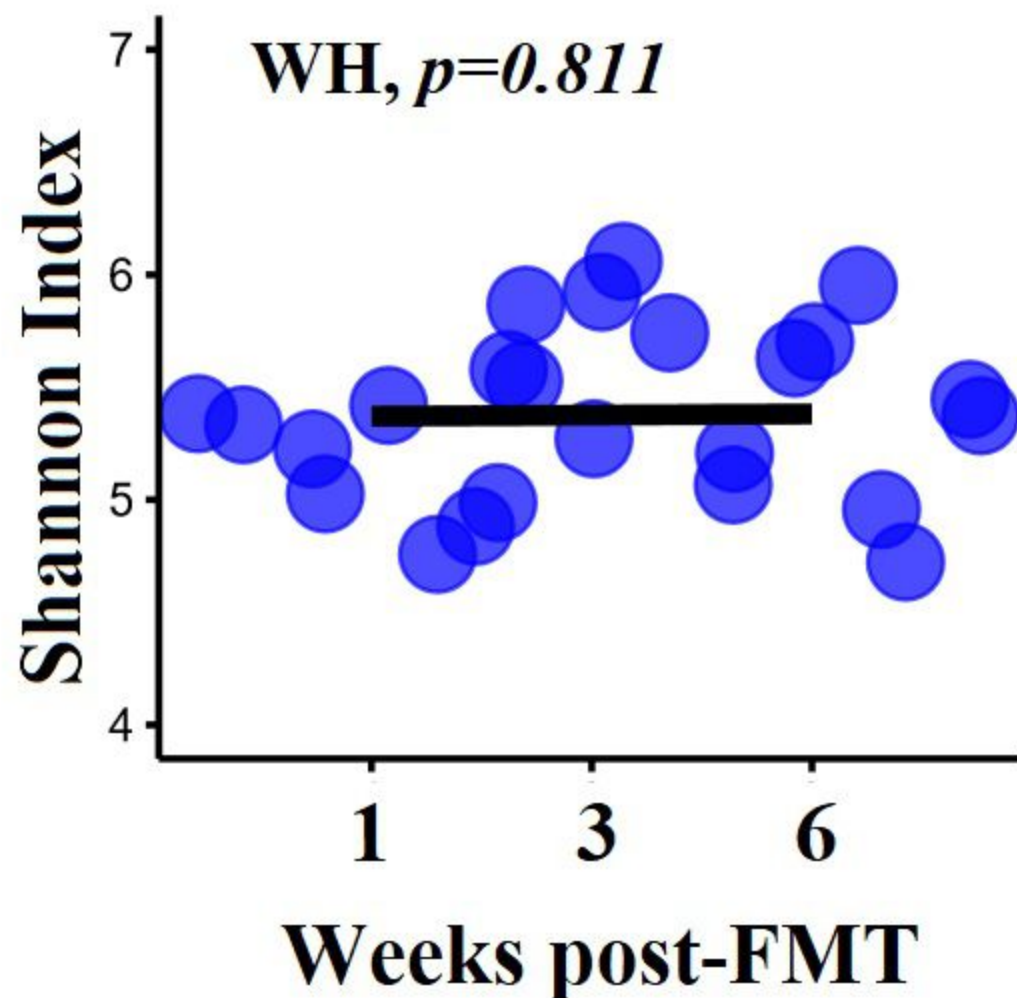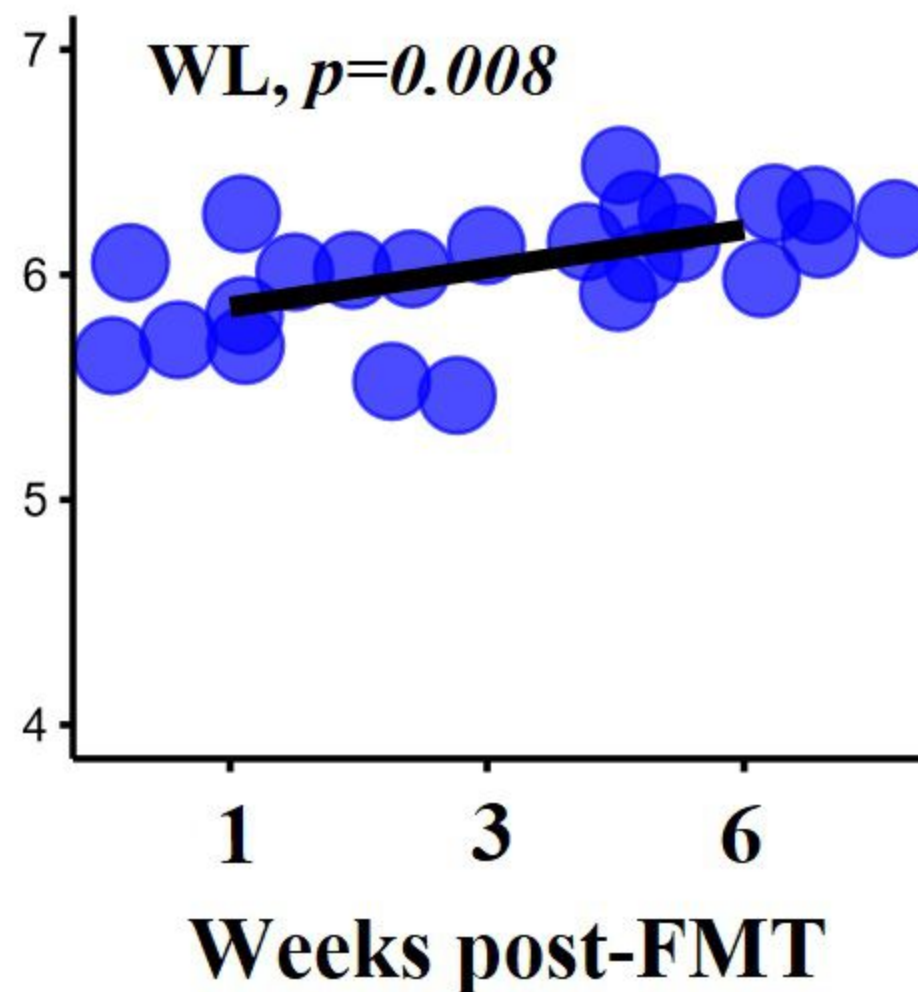

Supplement: Supplementary file 1 — Additional file 1:Figure S1. Shannon index for alpha diversity (evenness) 1 week, 3 weeks and 6 weeks after FMT. Shannon index significantly increased in the captive high, captive low, and wild low groups but remained stable in wild high, the leanest group. Linear regression and a groupwise average of the test statistic of the Spearman correlation were used to determine shifts within treatment groups. [file 42523_2020_33_MOESM1_ESM.pdf]

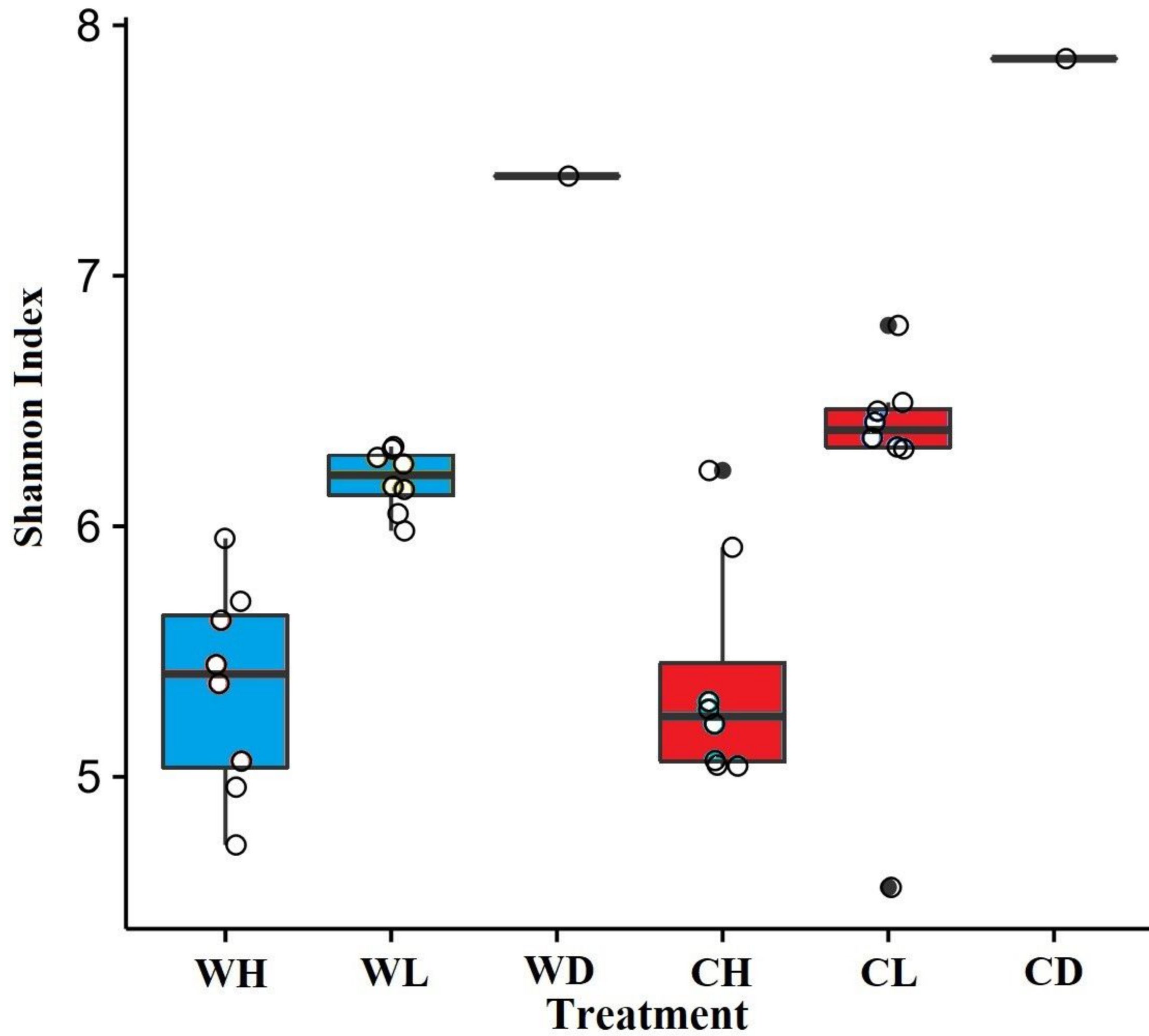

Supplement: Supplementary file 2 — Additional file 2: Figure S2. Shannon index for alpha diversity (evenness) in each treatment group. High-fiber groups had significantly lower Shannon index than low-fiber groups (p = 2.48e-14, ANOVA, FDR adjusted), but differences between wild and captive FMTs were not statistically significant. [file 42523_2020_33_MOESM2_ESM.pdf]

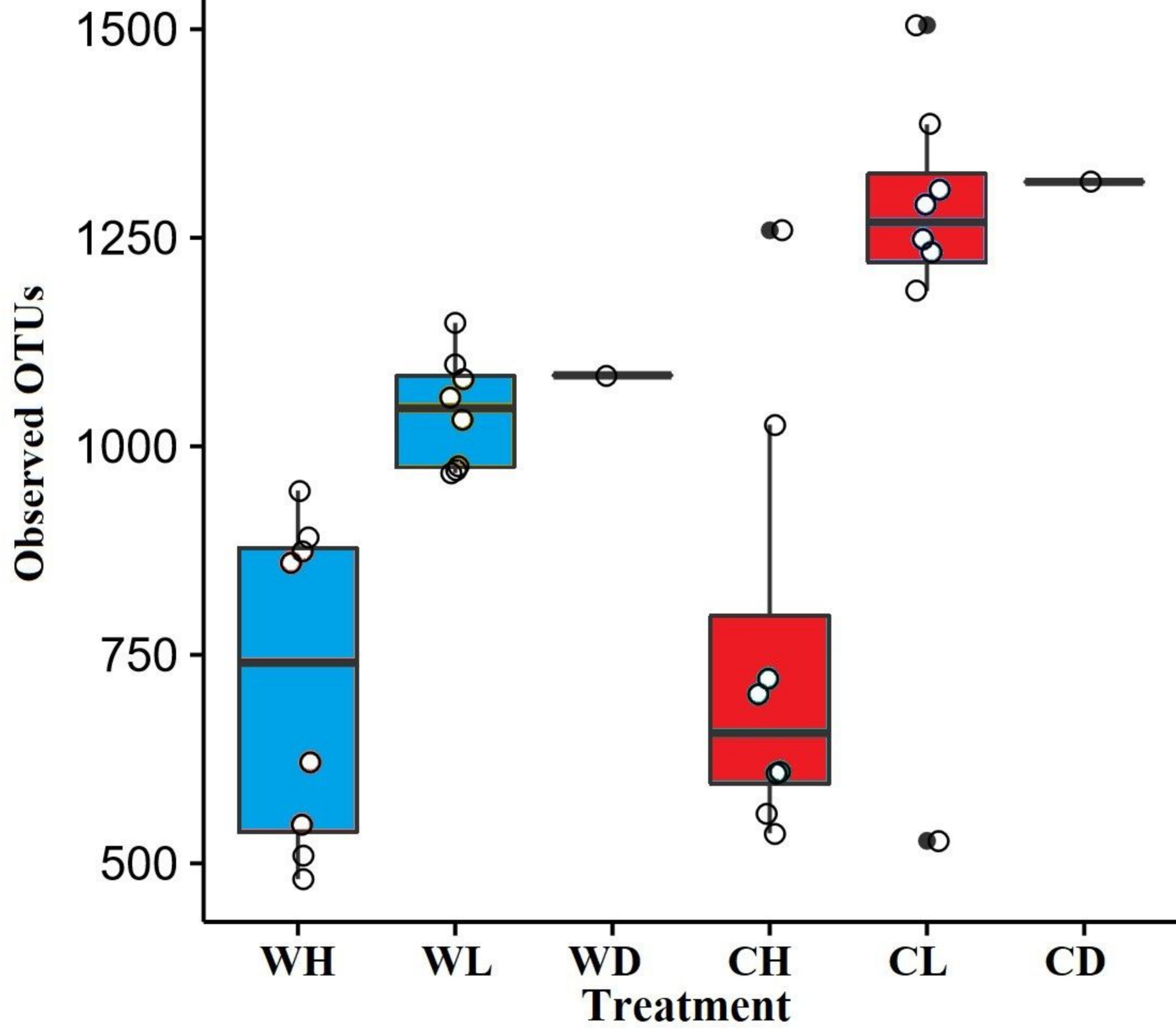

Supplement: Supplementary file 3 — Additional file 3: Figure S3. Observed OTUs in each treatment group. High-fiber groups had significantly lower observed OTUs than low-fiber groups (p = 2.69e-08, ANOVA, FDR adjusted), but differences between wild and captive FMTs were not statistically significant. [file 42523_2020_33_MOESM3_ESM.pdf]

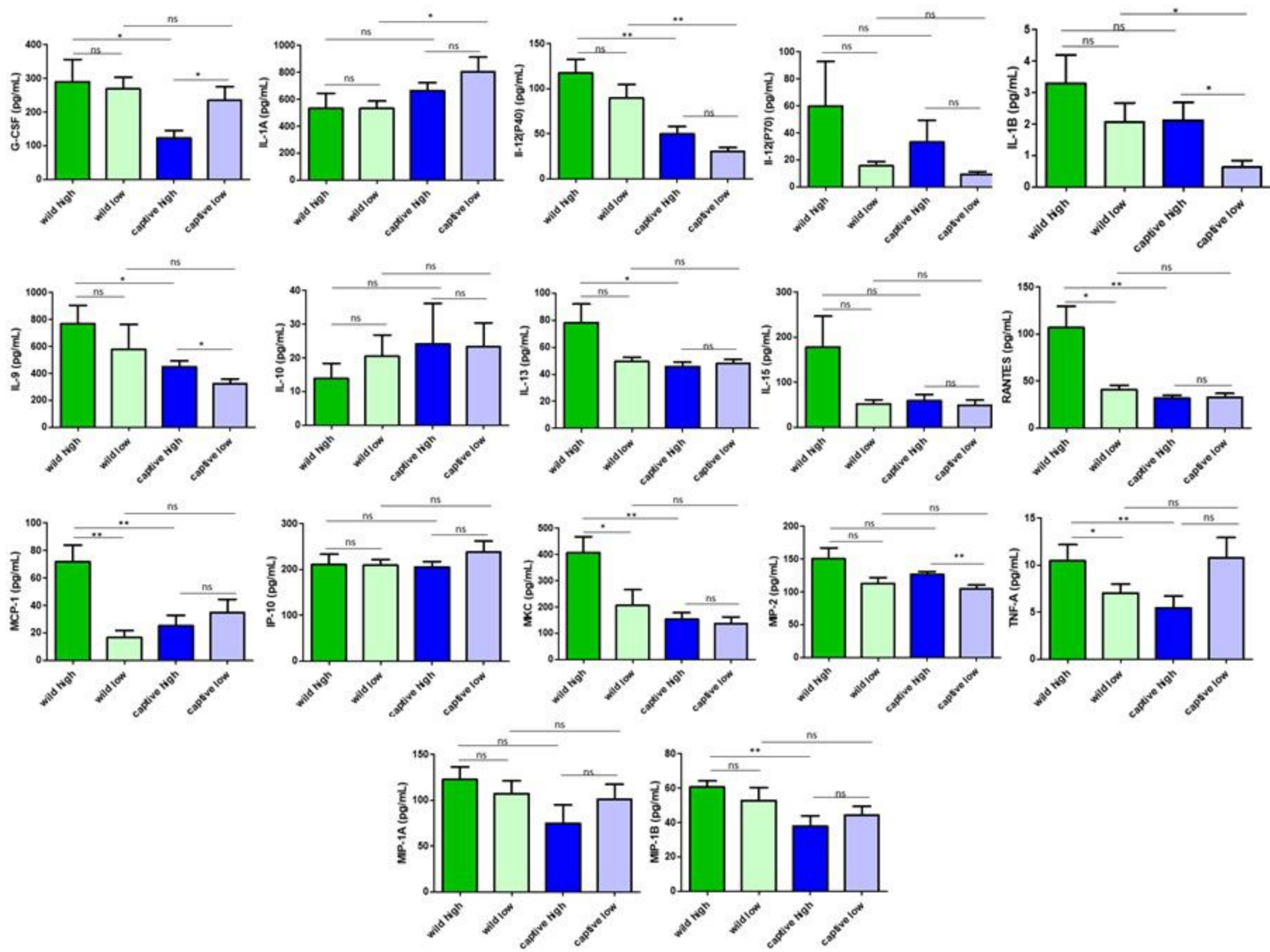

Supplement: Supplementary file 4 — Additional file 4: Figure S4. Detectable circulating blood cytokine and chemokine levels after sacrifice, from top left to bottom right: G-CSF, IL-1A, IL12(P40), IL-12(P70), IL-1B, IL-9, IL-10, IL-13, IL-15, RANTES, MCP-1, IP-10, MKC, MIP-2, TNF-A, MIP-1A, MIP-1B. All concentrations calculated in pg/mL Bars in green are showing WH, in light green WL, in blue CH and in purple CL. Concentrations of GM-CSF, IFN-G, IL-2, IL-4, IL-5, IL-6, IL-7 and IL-17 in most samples were lower than detectable level. Two-tailed t-test used (ns: no significant; * p < 0.05; ** p < 0.01; *** p < 0.001). [file 42523_2020_33_MOESM4_ESM.pdf]
