# Supplementary material for: Wild primate microbiomes prevent weight gain in germ-free mice
Source: Anim Microbiome. 2020 May 7;2:16. doi: 10.1186/s42523-020-00033-9 (PMC7807445; doi:10.1186/s42523-020-00033-9)
Supplement: Supplementary file 5 — Additional file 5:Figure S5. Low-fiber diet composition by manufacturer. [file 42523_2020_33_MOESM5_ESM.pdf]

TD.86489 Diet with Adjusted Sucrose/Corn Starch

Formula

Formula	g/Kg
Casein	207.0
DL-Methionine	3.0
Sucrose	320.99
Corn Starch	320.0
Corn Oil	50.0
Cellulose	50.0
Mineral Mix, AIN-76 (170915)	35.0
Calcium Carbonate	4.0
Vitamin Mix, Teklad (40060)	10.0
Ethoxyquin, antioxidant	0.01

Footnote

A diet with approximately equal amounts (wt/wt) of sucrose and corn starch. About 53% of the carbohydrate kcal come from sucrose, and 47% from corn starch. The pellets are relatively short due to the 1:1 ratio of corn starch and sucrose.

Selected Nutrient Information[1]

	% by weight	% kcal from
**Protein**	18.3	20.0
**Carbohydrate**	61.7	67.3
**Fat**	5.2	12.8

Kcal/g 3.7

[1] Values are calculated from ingredient analysis or manufacturer data

Teklad Diets are designed & manufactured for research purposes only.

Speak With A Nutritionist

- **(800) 483·5523**
- **askanutritionist@harlan.com**

Harlan Laboratories · PO Box 44220 · Madison, WI 53744-4220

www.harlan.com

Key Features

- **Purified Diet**
- **Adjusted Carbohydrate**
- **Sucrose & Corn Starch**
- **Rodent**

Key Planning Information

- *Products are made fresh to order*
- Store product at 4°C or lower
- Use within 6 months (applicable to most diets)
- Box labeled with product name, manufacturing date, and lot number
- Replace diet at minimum once per week

  More frequent replacement may be advised
- Lead time:
  - 2 weeks non-irradiated
  - 4 weeks irradiated

Product Specific Information

- 1/2" Pellet or Powder (free flowing)
- Minimum order 3 Kg
- Irradiation available upon request

Options (Fees Will Apply)

- Rush order (pending availability)
- Irradiation (see Product Specific Information)
- Vacuum packaging (1 and 2 Kg)

International Inquiry

·Outside U.S.A. or Canada·

- **askanutritionist@harlan.com**

Place Your Order (U.S.A. & Canada)

· Place Order · Obtain Pricing ·

· Check Order Status ·

- **(800) 483·5523**
- **(608) 277·2066** *facsimile*
- **tekladinfo@harlan.com**

Helping you do research better

Harlan, Harlan Laboratories, Helping you do research better,
and the Harlan logo are trademarks and trade names of Harlan Laboratories, Inc.
© 2008 Harlan Laboratories, Inc.
