# Supplementary material for: Wild primate microbiomes prevent weight gain in germ-free mice
Source: Anim Microbiome. 2020 May 7;2:16. doi: 10.1186/s42523-020-00033-9 (PMC7807445; doi:10.1186/s42523-020-00033-9)
Supplement: Supplementary file 6 — Additional file 6:Figure S6. High-fiber diet composition by manufacturer. [file 42523_2020_33_MOESM6_ESM.pdf]

Pico-Vac® Lab Rodent Diet 5061*

DESCRIPTION

Pico-Vac® Lab Rodent Diet is a 20% protein diet formulated for rat, hamster and mouse breeding colonies. This diet is a complete life cycle diet formulated using managed formulation, delivering Constant Nutrition®. This is paired with the selection of highest quality ingredients to assure minimal inherent biological variation in long-term studies. Irradiation treatment and special 4-ply packaging provide a virtually bacteria-free diet. The vacuum package provides a visual confirmation that the seal on the bag has not been broken.

Features and Benefits

- Managed Formulation delivers Constant Nutrition®
- Formulated with 20% protein for breeding colonies
- High quality animal protein added to create a superior balance of amino acids for optimum performance
- Recommended for rat breeding colonies and mice not requiring a higher energy diet
- Irradiation gives reliable microbial control and eliminates the need for autoclaving
- Precision processing and selection of highest quality ingredients assures Constant Nutrition® quality
- Vacuum packaged in small quantities (2.3 kg/5 lb) for ease of handling and to provide a visual check for package integrity

Product Forms Available

- Oval pellet, 10 mm x 16 mm x 25 mm length (3/8"x5/8"x1")
- Meal (ground pellets), special order

Other Versions Available

- 5053 PicoLab® Rodent Diet 20

GUARANTEED ANALYSIS

Crude protein not less than	20.0%
Crude fat not less than	4.5%
Crude fiber not more than	6.0%
Ash not more than	7.0%

INGREDIENTS

Ground corn, dehulled soybean meal, wheat middlings, ground wheat, fish meal, cane molasses, wheat germ, dried beet pulp, brewers dried yeast, dehydrated alfalfa meal, ground oats, soybean oil, dried whey, calcium carbonate, salt, DL-methionine, menadione dimethylpyrimidinol bisulfite (vitamin K), choline chloride, pyridoxine hydrochloride, cholecalciferol, vitamin A acetate, dl-alpha tocopheryl acetate, biotin, thiamine mononitrate, folic acid, vitamin B_{12} supplement, nicotinic acid, riboflavin, calcium pantothenate, manganous oxide, zinc oxide, ferrous carbonate, copper sulfate, zinc sulfate, calcium iodate, cobalt carbonate, sodium selenite.

FEEDING DIRECTIONS

Feed ad libitum to rodents. Plenty of fresh, clean water should be available to the animals at all times.

Rats- All rats will eat varying amounts of feed depending on their genetic origin. Larger strains will eat up to 30 grams per day. Smaller strains will eat up to 15 grams per day. Feeders in rat cages should be designed to hold two to three days supply of feed at one time.

Mice-Adult mice will eat up to 5 grams of pelleted ration daily. Some of the larger strains may eat as much as 8 grams per day per animal. Feed should be available on a free choice basis in wire feeders above the floor of the cage.

Hamsters-Adults will eat up to 14 grams per day.

For information regarding shelf life please visit www.labdiet.com.

12/11/09

CHEMICAL COMPOSITION[1]

Nutrients[2]

**Protein, %**	**20.0**
Arginine, %	1.22
Cystine, %	0.28
Glycine, %	0.96
Histidine, %	0.50
Isoleucine, %	0.97
Leucine, %	1.56
Lysine, %	1.16
Methionine, %	0.70
Phenylalanine, %	0.90
Tyrosine, %	0.59
Threonine, %	0.77
Tryptophan, %	0.26
Valine, %	1.00
Serine, %	1.03
Aspartic Acid, %	2.19
Glutamic Acid, %	4.34
Alanine, %	1.15
Proline, %	1.47
Taurine, %	0.02
**Fat (ether extract), %**	**5.0**
**Fat (acid hydrolysis), %**	**5.6**
Cholesterol, ppm	141
Linoleic Acid, %	2.19
Linolenic Acid, %	0.26
Arachidonic Acid, %	<0.01
Omega-3 Fatty Acids, %	0.33
Total Saturated Fatty Acids, %	0.93
Total Monounsaturated Fatty Acids, %	0.99
**Fiber (Crude), %**	**4.7**
Neutral Detergent Fiber[3], %	16.4
Acid Detergent Fiber[4], %	6.0
**Nitrogen-Free Extract (by difference), %**	**52.9**
Starch, %	33.9
Glucose, %	0.19
Fructose, %	0.23
Sucrose, %	3.18
Lactose, %	1.34
**Total Digestible Nutrients,%**	**76.2**
**Gross Energy, kcal/gm**	**4.07**
**Physiological Fuel Value[5], kcal/gm**	**3.41**
**Metabolizable Energy, kcal/gm**	**3.07**

Minerals

**Ash, %**	**6.1**
Calcium, %	0.81
Phosphorus, %	0.63
Phosphorus (non-phytate), %	0.33
Potassium, %	1.07
Magnesium, %	0.22
Sulfur, %	0.34
Sodium, %	0.30
Chloride, %	0.51
Fluorine, ppm	10
Iron, ppm	220
Zinc, ppm	87
Manganese, ppm	85
Copper, ppm	13
Cobalt, ppm	0.71
Iodine, ppm	0.97
Chromium, ppm	0.81
Selenium, ppm	0.30

Vitamins

Carotene, ppm	1.5
Vitamin K (as menadione),ppm	3.3
Thiamin Hydrochloride, ppm	17
Riboflavin, ppm	8.0
Niacin, ppm	90
Pantothenic Acid, ppm	17
Choline Chloride, ppm	2000
Folic Acid, ppm	3.0
Pyridoxine, ppm	9.6
Biotin, ppm	0.30
B_{12}, mcg/kg	51
Vitamin A, IU/gm	15
Vitamin D_3 (added), IU/gm	2.2
Vitamin E, IU/kg	99
Ascorbic Acid, mg/gm	—

Calories provided by:

Protein, %	24.651
Fat (ether extract), %	13.205
Carbohydrates, %	62.144

*Product Code

1. Formulation based on calculated values from the latest ingredient analysis information. Since nutrient composition of natural ingredients varies and some nutrient loss will occur due to manufacturing processes, analysis will differ accordingly.
2. Nutrients expressed as percent of ration except where otherwise indicated. Moisture content is assumed to be 10.0% for the purpose of calculations.
3. NDF = approximately cellulose, hemi-cellulose and lignin.
4. ADF = approximately cellulose and lignin.
5. Physiological Fuel Value (kcal/gm) = Sum of decimal fractions of protein, fat and carbohydrate (use Nitrogen Free Extract) x 4,9,4 kcal/gm respectively.

LabDiet®
www.labdiet.com
