# Supplementary material for: Wild primate microbiomes prevent weight gain in germ-free mice
Source: Anim Microbiome. 2020 May 7;2:16. doi: 10.1186/s42523-020-00033-9 (PMC7807445; doi:10.1186/s42523-020-00033-9)
Supplement: Supplementary file 7 — Additional file 7:Table S1. Bacterial taxa in donor FMTs that are missing in their respective treatment groups. [file 42523_2020_33_MOESM7_ESM.pdf]

Captive FMT Genera		Wild FMT Genera	
Holdemania	*Williamsia*	*Acinetobacter*	*Gemmatimonas*
Coprobacillus	*Micrococcus*	*Anaerococcus*	*Haemophilus*
Lachnospira	*Bilophila*	*Anaerotruncus*	*Janibacter*
Mogibacterium	*Anaerovibrio*	*Atopobium*	*Janthinobacterium*
Treponema	*Sphaerochaeta*	*Bdellovibrio*	*Kaistobacter*
Phascolarctobacterium	*Parvimonas*	*Brachybacterium*	*Kytococcus*
Faecalibacterium	*Oxalobacter*	*Burkholderia*	*Lachnobacterium*
CF231	*Selenomonas*	*Butyrivibrio*	*Leadbetterella*
Sutterella	*Pyramidobacter*	*Campylobacter*	*Methylocaldum*
Methanosphaera	*Sharpea*	*Candidatus Nitrososphaera*	*Mycobacterium*
Corynebacterium	*Syntrophomonas*	*Candidatus Xiphinematobacter*	*Nitrospira*
YRC22	*K82*	*Chryseobacterium*	*Nocardioides*
Pseudomonas		*Conexibacter*	*Pedomicrobium*
Butyrivibrio		*Corynebacterium*	*Peptoniphilus*
p-75-a5		*Cupriavidus*	*Pseudonocardia*
Dechloromonas		*DA101*	*Rhodobacter*
vadinCA11		*Dehalobacterium*	*Rhodoplanes*
Slackia		*Enhydrobacter*	*Rothia*
Atopobium		*Erwinia*	*Sphingobacterium*
Bulleidia		*Faecalibacterium*	*Synechococcus*
Elusimicrobium		*Finegoldia*	*Tepidimonas*
Actinomyces		*Flavobacterium*	*Tsukamurella*
Microbispora		*Fluviicola*	*YRC22*
