# Supplementary material for: Wild primate microbiomes prevent weight gain in germ-free mice
Source: Anim Microbiome. 2020 May 7;2:16. doi: 10.1186/s42523-020-00033-9 (PMC7807445; doi:10.1186/s42523-020-00033-9)
Supplement: Supplementary file 8 — Additional file 8:Table S2. Microbiota transfer efficacy as indicated by the total bacterial species & mean observed OTUs identified in fecal samples collected on day 49 and compared to donor FMT. [file 42523_2020_33_MOESM8_ESM.pdf]

		Species Recovered	Treatment /FMT (Species ratio)	*Mean* OTUs	Treatment /FMT (OTUs ratio)
Treatment	**WH**	162	0.74	716	0.66
	**WL**	172	0.78	1042	0.96
	**CH**	147	0.91	753	0.57
	**CL**	175	1.08	1210	0.92
FMT	**WD**	220	--	1085	--
	**CD**	162	--	1317	--
